# Supplementary material for: Role of the Maritime Continent in the remote influence of Atlantic Niño on the Pacific
Source: Nat Commun. 2023 Jun 7;14:3327. doi: 10.1038/s41467-023-39036-w (PMC10247805; doi:10.1038/s41467-023-39036-w)
Supplement: Supplementary file 1 — Supplementary Information [file 41467_2023_39036_MOESM1_ESM.pdf]

Supplementary Information for

Role of the Maritime Continent in the remote influence of Atlantic Niño on the Pacific

Siying Liu[1,2], Ping Chang[3]*, Xiuquan Wan[1,2]*, Stephen G. Yeager[4] & Ingo Richter[5]

*Corresponding authors. E-mails: ping@tamu.edu; xqwan@ouc.edu.cn

Title of file for HTML: Supplementary Information

Description: Supplementary Figures, Supplementary Tables and Supplementary Discussion

Supplementary Figures

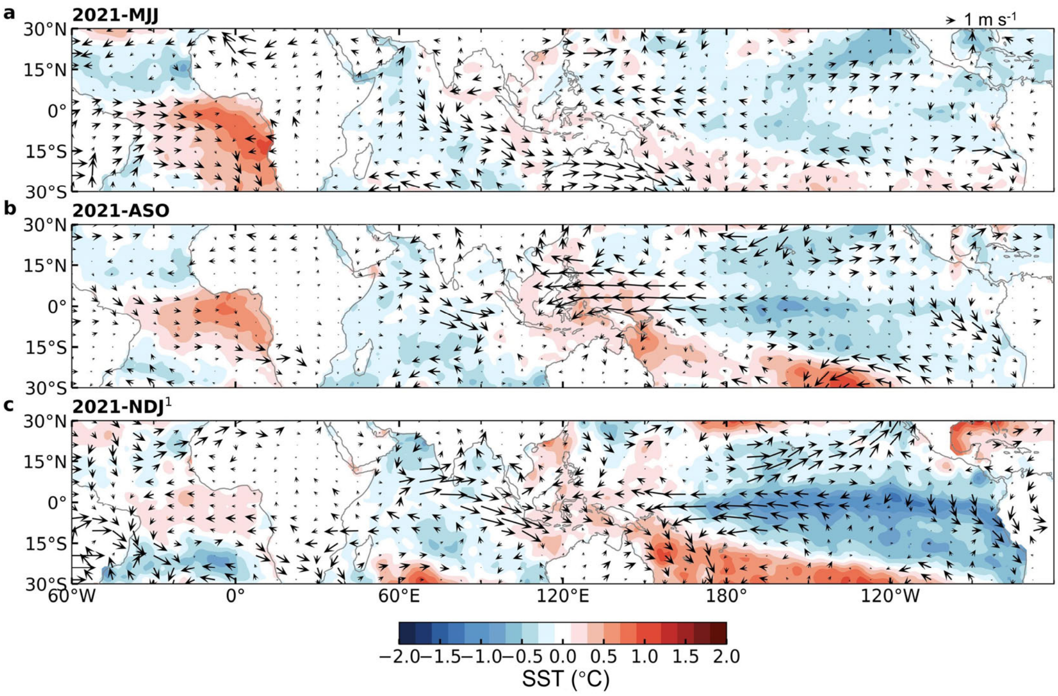

Supplementary Fig 1. **The Atlantic Niño and Pacific La Niña events during 2021-2022 in reanalysis and observation. a-c** Detrended Sea Surface Temperature (SST) (°C; color shading) anomalies and wind anomalies at 850 hPa (m s^{-1}; arrows) over the boreal summer (May-June-July (MJJ)) (**a**), fall (August-September-October (ASO)) (**b**) and winter (November-December-January (NDJ^1)) (**c**) in 2021-2022 from HadISST and ERA5 Reanalysis respectively. Source data are provided as a Source Data file in the Zenodo database.

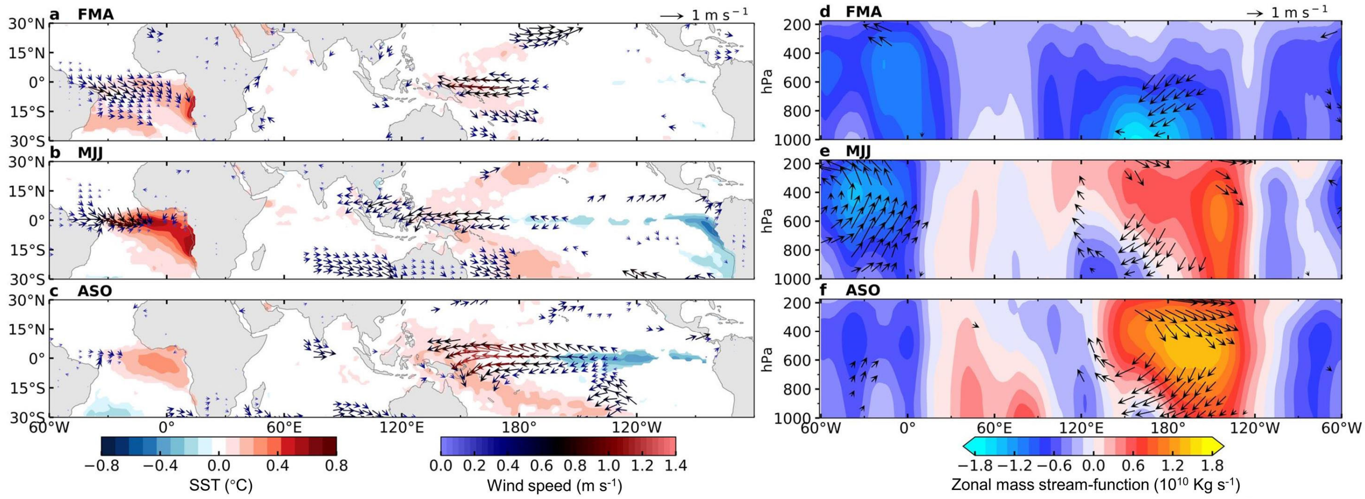

Supplementary Fig 2. **Observed tropical Sea Surface Temperature and Walker Circulation anomalies response to Atlantic Niño excluding the strong El Niño-Southern Oscillation years.** **a-c** Boreal spring (February-March-April (FMA)) (**a**), summer (May-June-July (MJJ)) (**b**), and fall (August-September-October (ASO)) (**c**) Sea Surface Temperature (SST) (°C; color shading) anomalies (HadISST datasets, 1959-2021) and wind anomalies at 1000hPa (m s^{-1}; color arrows) averaged by three reanalysis datasets (ERA5, JRA55 for 1959-2021 and ERA-Interim for 1979-2018) regressed onto the normalized May-June-July (MJJ) Atlantic Niño-related variability (ATL3 index) after first excluding the years of strong El Niño-Southern Oscillation from the data record (Methods). **d-f** Same as **a-c** except for zonal and vertical wind anomalies (m s^{-1}; arrows; vertical winds multiplied by 100.) averaged from 5°S to 5°N and zonal mass stream-function anomalies (Kg s^{-1}; color shading; see Methods) at 175-1000 hPa pressure levels. The colors (Fig a-c) and vectors (Fig a-f) indicate statistically significant at 10% level based on a two-sided Student's t-test. The statistical test was not performed for the zonal mass stream-function because its main purpose is to illustrate the direction of the zonal circulations. Source data are provided as a Source Data file in the Zenodo database.

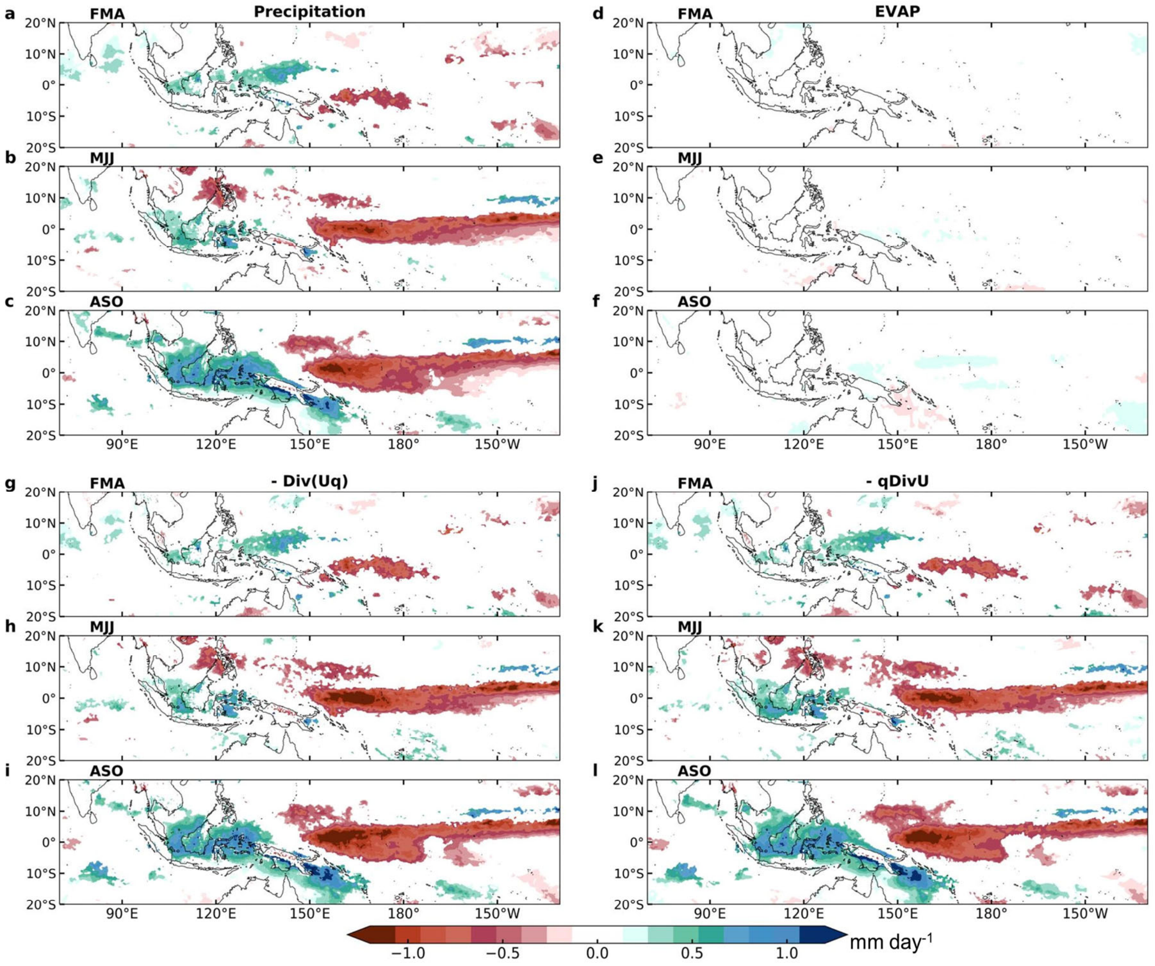

Supplementary Fig 3. **Response of precipitation and moisture-related terms in the Maritime Continent-Western Pacific region to Atlantic Niño in reanalysis on seasonal timescales. a-c** Boreal spring (February-March-April (FMA)) (**a**), summer (May-June-July (MJJ)) (**b**), and fall (August-September-October (ASO)) (**c**) precipitation (mm day^{-1}; color shading) anomalies in reanalysis datasets (ERA5, 1959-2021) regression against the normalized May-June-July (MJJ) Atlantic Niño-related variability (ATL3 index) after linearly regressing out El Niño-Southern Oscillation related variability from the data record (Methods). **d-f** Same as **a-c** except for evaporation (mm day^{-1}; color shading; referred as EVAP). **g-i** Same as **a-c** except for vertical integral of divergence of moisture flux multiplied by -1 (mm day^{-1}; color shading; referred as -Div(Uq)). **j-l** Same as **a-c** except for vertically integrated moisture divergence multiplied by -1 (mm day^{-1}; color shading; referred as -qDivU). Source data are provided as a Source Data file in the Zenodo database.

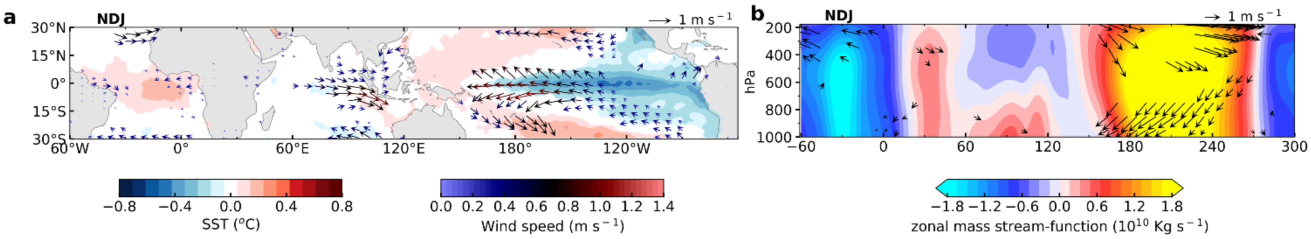

Supplementary Fig 4. **Observed tropical Sea Surface Temperature and Walker Circulation anomalies response to Atlantic Niño in the boreal winter. a** Boreal winter November-December-January (NDJ) Sea Surface Temperature (SST) (°C; color shading) anomalies (HadISST datasets, 1959-2021) and wind anomalies at 1000hPa (m s^{-1}; color arrows) averaged using three reanalysis datasets (ERA5, JRA55 for 1959-2021 and ERA-Interim for 1979-2018) and regressed onto the normalized May-June-July (MJJ) Atlantic Niño-related variability (ATL3 index) after linearly regressing out (December-January-February (DJF)) El Niño-Southern Oscillation related variability (Niño3.4 index). **b** Same as **a** except for zonal and vertical wind anomalies (m s^{-1}; arrows; vertical winds multiplied by 100) averaged between 5°S and 5°N, and zonal mass stream-function anomalies (Kg s^{-1}; color shading; see Methods) from 175 to 1000 hPa pressure levels. The colors (Fig a) and vectors (Fig a-b) indicate statistical significance at the 10% level based on a two-sided Student's t-test. The statistical test was not performed for the zonal mass stream-function because its main purpose is to illustrate the direction of the zonal circulation anomalies. Source data are provided as a Source Data file in the Zenodo database.

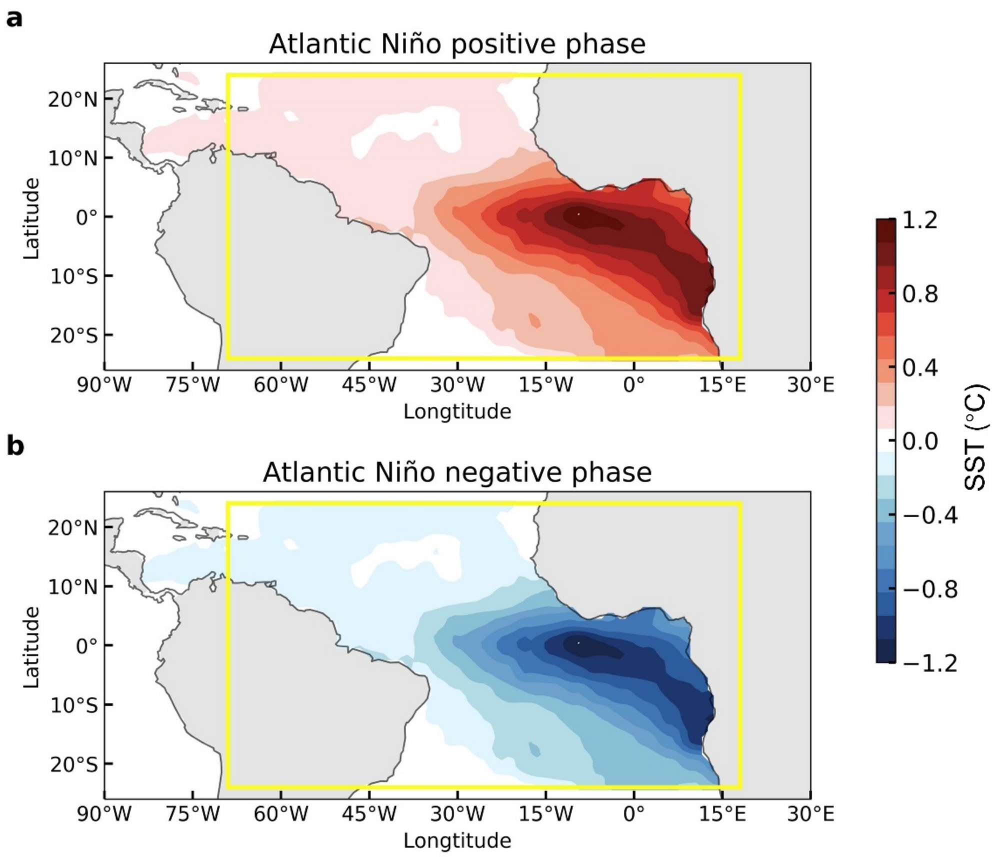

Supplementary Fig 5. **Atlantic Niño Sea Surface Temperature anomalies spatial pattern derived from observation for the three large-ensembles. a-b** Sea Surface Temperature (SST) anomalies (°C; color shading) from HadISST datasets during 1870-2017 regressed onto the June-July-August (JJA) Atlantic Niño-related variability (ATL3 index) multiplied by +1 for the control experiment (CTRL) with positive Atlantic Niño SST anomalies forcing (CTRL+), the topography removal experiment (NTOP) with positive Atlantic Niño SST anomalies forcing (NTOP+) and the land-friction reduction experiment (NFRC) with positive Atlantic Niño SST anomalies forcing (NFRC+) described in the text (**a**) and by -1 for the CTRL simulation with negative Atlantic Niño SST anomalies forcing (CTRL-), NTOP- and NFRC- simulations (**b**) respectively. The yellow boxes in **a-b** show the region where SST restoring was used in the simulation. Source data are provided as a Source Data file in the Zenodo database.

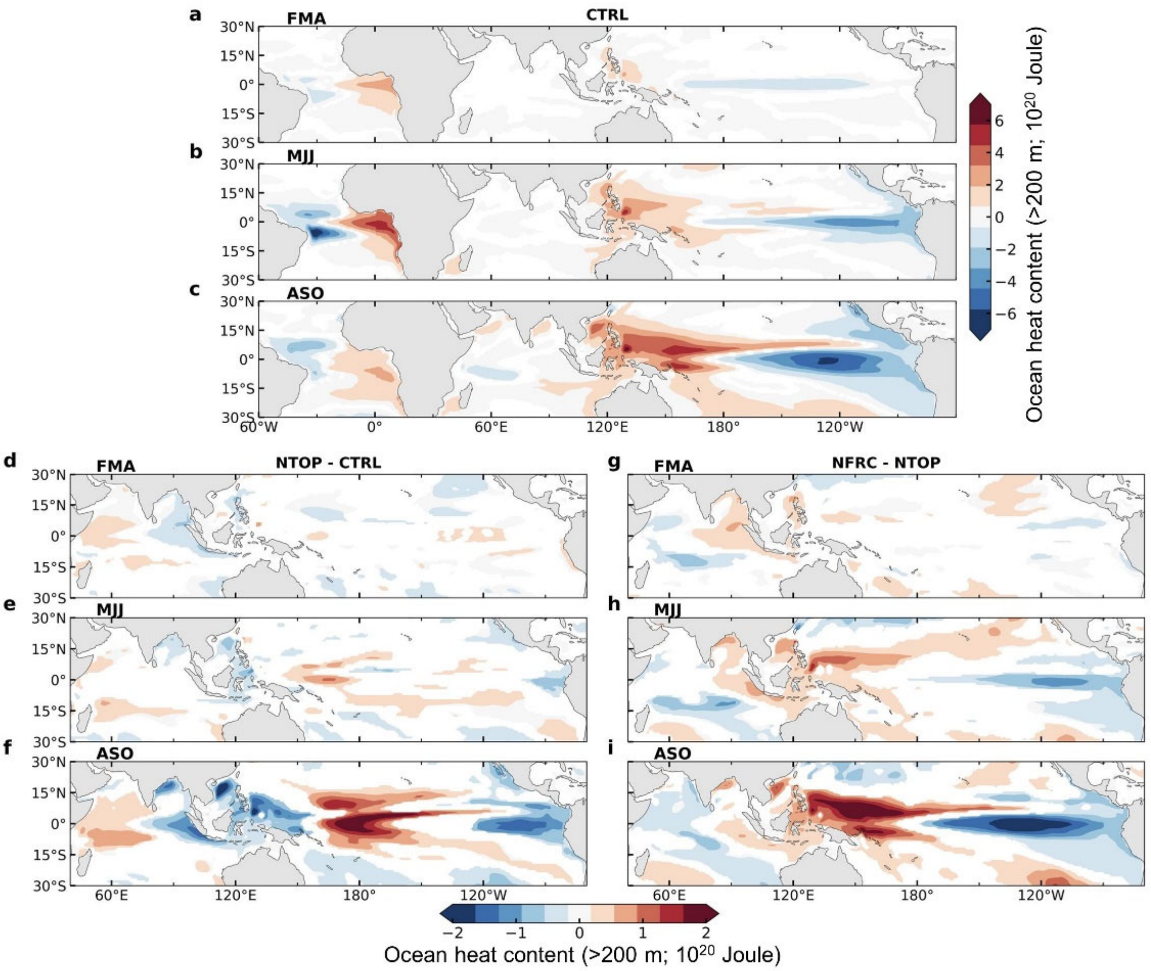

Supplementary Fig 6. **Simulated response of ocean heat content to Atlantic Niño Sea Surface Temperature (SST) in the three sets of large-ensemble experiments. a-c** Simulated ocean heat content (10^{20} Joule; color shading) in upper 200 meters averaged over the boreal spring (February-March-April (FMA)) (**a**), summer (May-June-July (MJJ)) (**b**) and fall (August-September-October (ASO)) (**c**) for the control experiment (CTRL) described in the text; **d-f** Same as **a-c** except for the anomalies in the topography removal experiment (NTOP) minus those in CTRL; **g-i** Same as **a-c** except for the anomalies in the land-friction reduction experiment (NFRC) minus those in NTOP. Anomalies in CTRL, NTOP and NFRC are obtained by CTRL with positive Atlantic Niño SST anomalies forcing minus CTRL with negative Atlantic Niño SST anomalies forcing (CTRL+ minus CTRL-), NTOP+ minus NTOP-, and NFRC+ minus NFRC-, respectively. Colors represent those that are significant at the 10% level based on a two-sided Student's t-test. Note that in **d-f** and **g-i** the tropical Atlantic sector is excluded because the forcing is the same for all three ensembles. Source data are provided as a Source Data file in the Zenodo database.

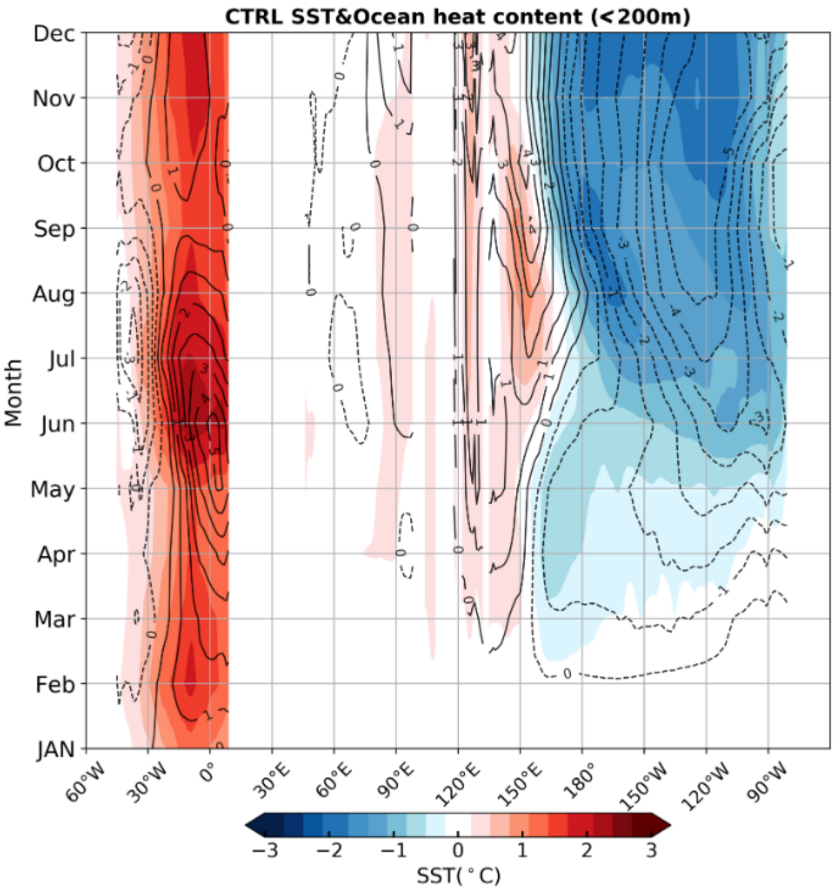

Supplementary Fig 7. **Simulated dynamical adjustment of the equatorial ocean response to Atlantic Niño in the large-ensemble experiments.** Hovmöller diagram of Sea Surface Temperature (SST) (°C; color shading) and ocean heat content (10^{20} Joule; contours) anomalies in upper 200 meters in the control experiment (CTRL) described in the text (CTRL with positive Atlantic Niño SST anomalies forcing minus CTRL with negative Atlantic Niño SST anomalies forcing (CTRL+ minus CTRL-)) from January to December averaged from 2°S to 2°N along the equator. Source data are provided as a Source Data file in the Zenodo database.

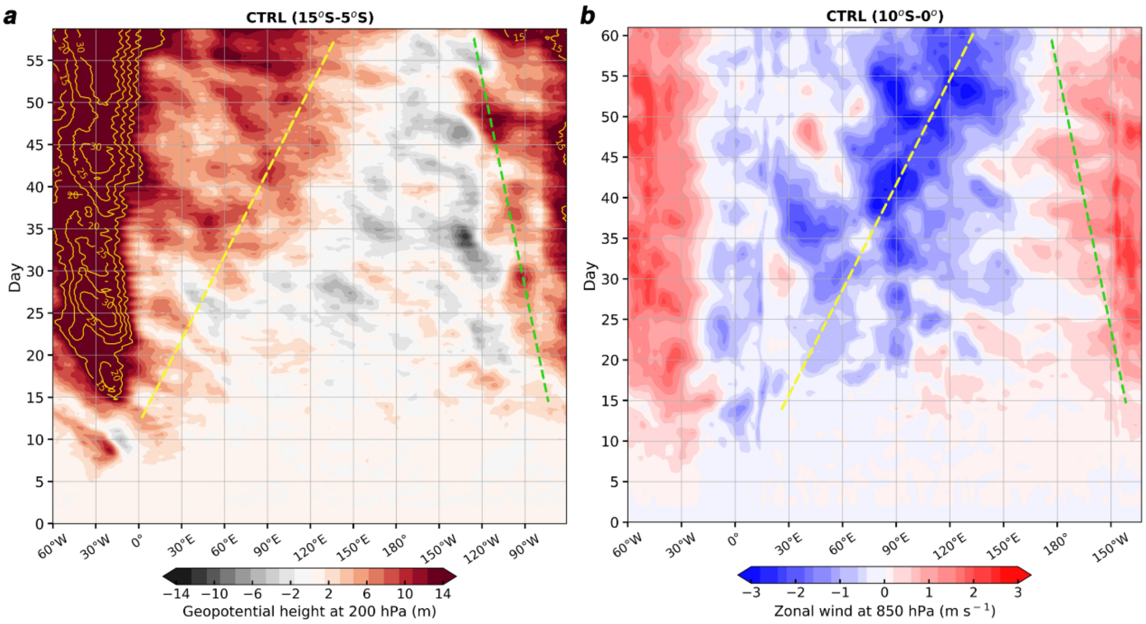

Supplementary Fig 8. **Simulated atmospheric Kelvin wave-like and Rossby wave-like responses in the large-ensemble experiments. a-b** Hovmöller diagrams of the simulated meridional mean geopotential height at 200 hPa six-hourly anomalies (**a**) (m; color shading and contours) averaged from 15°S to 5°S and zonal wind daily anomalies at 850 hPa (**b**) (m s^{-1}; color shading) averaged from 10°S to 0° along the equator by the control experiment (CTRL) described in the text with positive Atlantic Niño SST anomalies forcing minus CTRL with negative Atlantic Niño SST anomalies forcing (CTRL+ minus CTRL-). The yellow lines indicate the eastward propagating atmospheric Kelvin wave. The green lines indicate the westward propagating atmospheric Rossby wave. Source data are provided as a Source Data file in the Zenodo database.

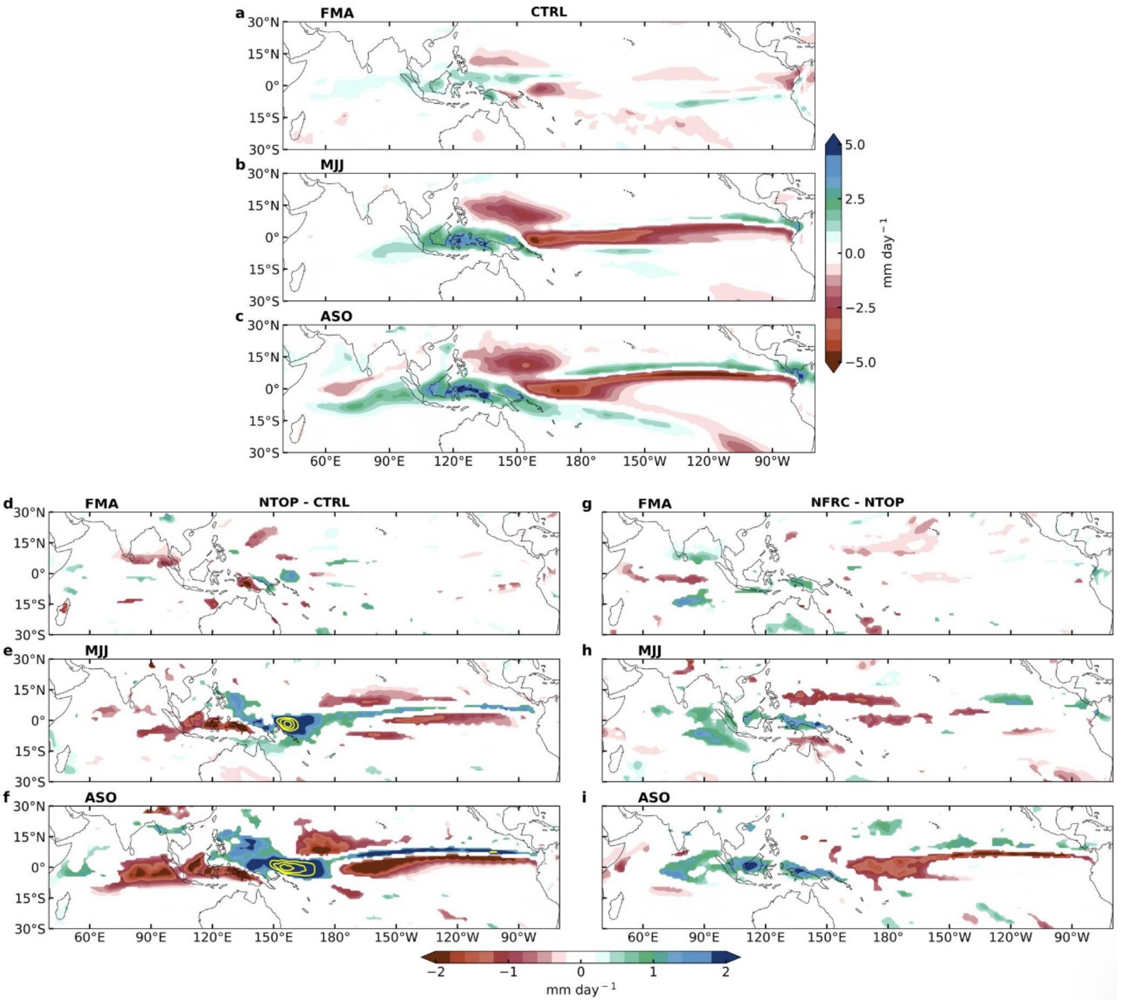

Supplementary Fig 9. **Simulated response of precipitation to Atlantic Niño Sea Surface Temperature (SST) in the three sets of large-ensemble experiments. a-c** Simulated precipitation (mm day^{-1}; color shading) averaged over boreal spring (February-March-April (FMA)) (**a**), summer (May-June-July (MJJ)) (**b**) and fall (August-September-October (ASO)) (**c**) for the control experiment (CTRL) described in the text. **d-f** Same as **a-c** except for the anomalies in the topography removal experiment (NTOP) minus those in CTRL. The heavy differences of precipitation in **d-f** are indicated by the yellow contour lines at 3, 4, 5 mm day^{-1}. **g-i** Same as **a-c** except for the anomalies in the land-friction reduction experiment (NFRC) minus those in NTOP. Anomalies in CTRL, NTOP and NFRC are obtained by CTRL with positive Atlantic Niño SST anomalies forcing minus CTRL with negative Atlantic Niño SST anomalies forcing (CTRL+ minus CTRL-), NTOP+ minus NTOP-, and NFRC+ minus NFRC-, respectively. Colors represent those that are significant at the 10% level based on a two-sided Student's t-test. Note that in **d-f** and **g-i** the tropical Atlantic sector is excluded because the forcing is the same for all three ensembles. Source data are provided as a Source Data file in the Zenodo database.

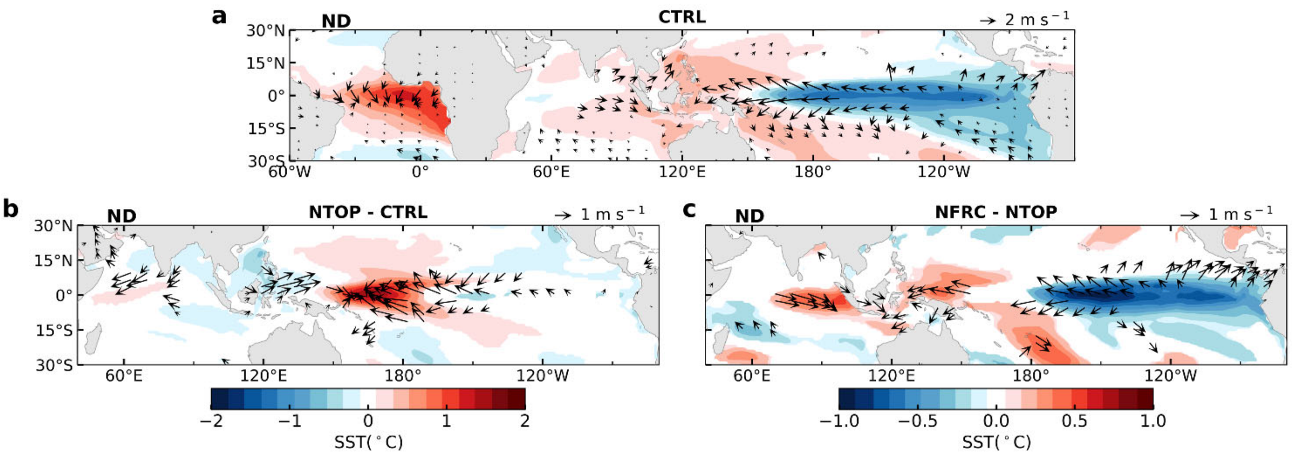

Supplementary Fig 10. **Simulated response of Sea Surface Temperature and low-level wind anomalies to Atlantic Niño Sea Surface Temperature in the three sets of large-ensemble experiments. a** Simulated Sea Surface Temperature (SST) (°C; color shading) and wind anomalies at 992 hPa (m s^{-1}; arrows) averaged over boreal winter November-December (ND) in the control experiment (CTRL) described in the text. **b** Same as **a** except for the anomalies in the topography removal experiment (NTOP) minus those in CTRL. **c** Same as **a** except for the anomalies in the land-friction reduction experiment (NFRC) minus those in NTOP. Anomalies in CTRL, NTOP and NFRC are obtained by taking ensemble mean of CTRL with positive Atlantic Niño SST anomalies forcing minus CTRL with negative Atlantic Niño SST anomalies forcing (CTRL+ minus CTRL-), ensemble mean of NTOP+ minus NTOP-, and ensemble mean of NFRC+ minus NFRC-, respectively. Colors and vectors represent those that are significant at the 10% level based on a two-sided Student's t-test. Note that in **b** and **c** the tropical Atlantic sector is excluded because the forcing is the same for all three experiments. Source data are provided as a Source Data file in the Zenodo database.

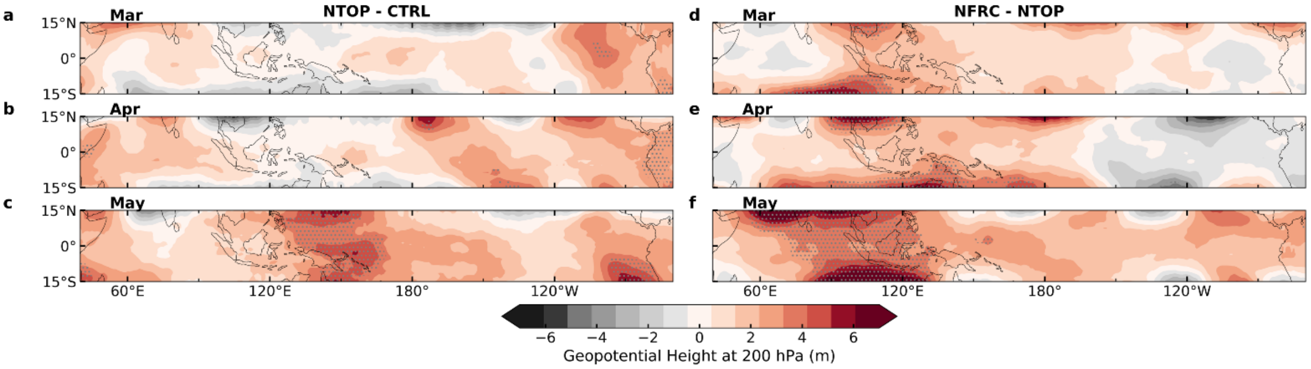

Supplementary Fig 11. **Simulated atmospheric Kelvin wave-like response in the large-ensemble experiments. a-c** Simulated geopotential height anomalies at 200 hPa (m; color shading) in March (Mar) (**a**), April (Apr) (**b**), May (**c**) for the topography removal experiment (NTOP) minus the control experiment (CTRL) described in the text. Pots represent those that are significant at the 10% level based on a two-sided Student's t-test. **d-f** Same as **a-c** except for the land-friction reduction experiment (NFRC) minus NTOP. Anomalies in CTRL, NTOP and NFRC are obtained by taking ensemble-mean of CTRL with positive Atlantic Niño SST anomalies forcing minus CTRL with negative Atlantic Niño SST anomalies forcing (CTRL+ minus CTRL-), ensemble-mean of NTOP+ minus NTOP-, and ensemble-mean of NFRC+ minus NFRC-, respectively. Source data are provided as a Source Data file in the Zenodo database.

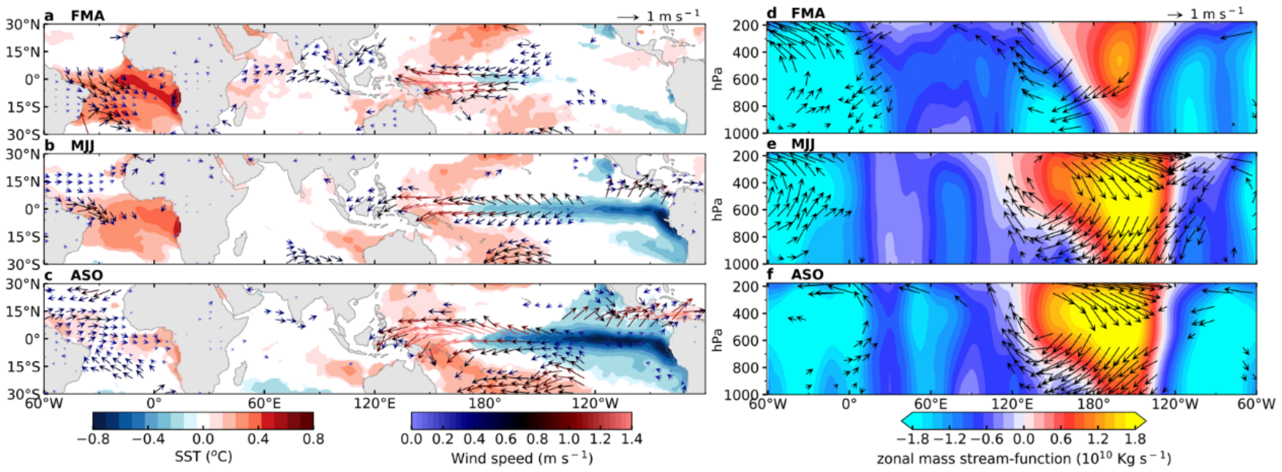

Supplementary Fig 12. **Observed tropical Sea Surface Temperature and Walker Circulation anomalies response to Atlantic Niño. a-c** Boreal spring (February-March-April (FMA)) (**a**), summer (May-June-July (MJJ)) (**b**), and fall (August-September-October (ASO)) (**c**) Sea Surface Temperature (SST) (°C; color shading) anomalies (HadISST datasets, 1959-2021) and wind anomalies at 1000hPa (m s^{-1}; color arrows) averaged using three reanalysis datasets (ERA5, JRA55 for 1959-2021 and ERA-Interim for 1979-2018) and regressed onto the normalized February-March-April (FMA) Atlantic Niño-related variability (ATL3 index) after linearly regressing out December-January-February (DJF) El Niño-Southern Oscillation related variability (Niño3.4 index). **d-f** Same as **a-c** except for zonal and vertical wind anomalies (m s^{-1}; arrows; vertical winds multiplied by 100) averaged between 5°S and 5°N, and zonal mass stream-function anomalies (Kg s^{-1}; color shading; see Methods) from 175 to 1000 hPa pressure levels. The colors (Fig a-c) and vectors (Fig a-f) indicate statistical significance at the 10% level based on a two-sided Student's t-test. The statistical test was not performed for the zonal mass stream-function because its main purpose is to illustrate the direction of the zonal circulation anomalies. Source data are provided as a Source Data file in the Zenodo database.

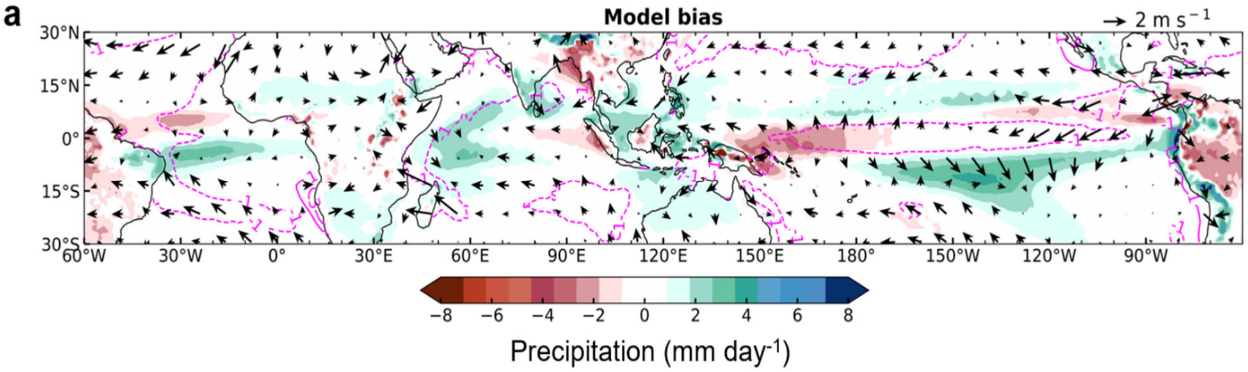

Supplementary Fig 13. **The Community Earth System Model version 1 (CESM1) model biases in the tropics. a** Simulated sea surface temperature (°C; contours), wind at 1000 hPa (m s^{-1}; arrows) and precipitation (mm day^{-1}; color shading) biases reference to HadISST datasets and ERA5 Reanalysis for 1959-2021. The model means are derived from a 2200-year preindustrial CESM1 simulation at nominal ~1°resolution for the period of year 1500 to year 1562 - the same 63-year period as in observations. Source data are provided as a Source Data file in the Zenodo database.

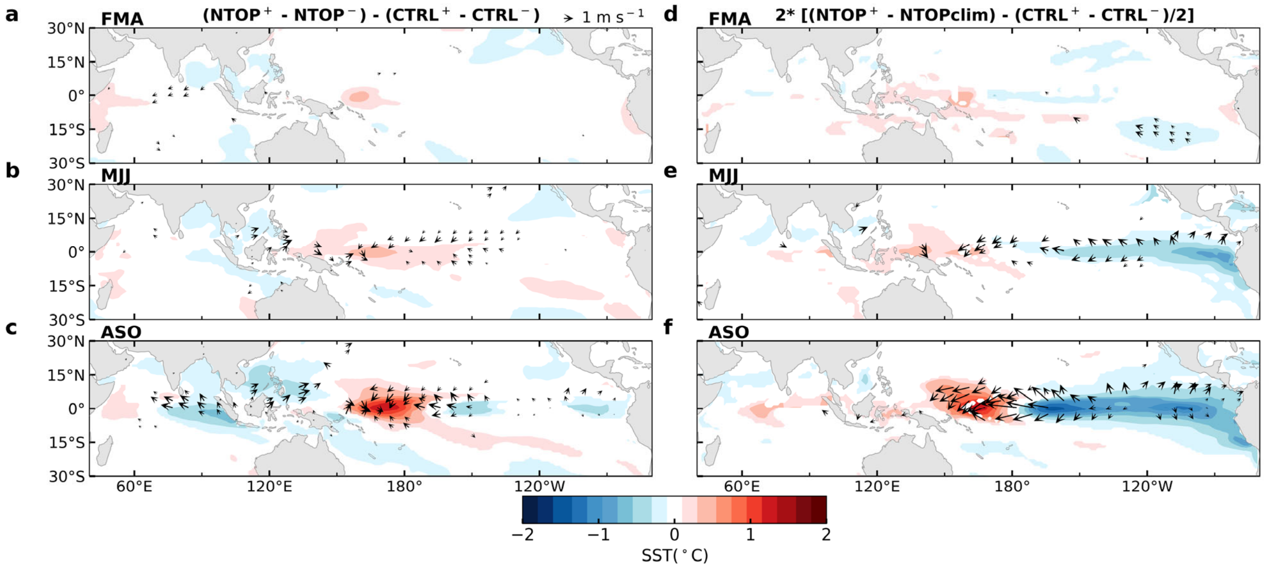

Supplementary Fig 14. **Assessment of the weak nonlinearity assumption in this study. a-c** Simulated Sea Surface Temperature (SST) (°C; color shading) and winds at 992 hPa (m s^{-1}; vectors) anomalies during boreal spring (February-March-April (FMA)) (**a**), summer (May-June-July (MJJ)) (**b**) and winter (August-September-October (ASO)) (**c**) respectively by the land-friction reduction experiment (NTOP) minus the control experiment (CTRL) described in the text. Anomalies in CTRL and NTOP are obtained by taking ensemble-mean of CTRL with positive Atlantic Niño SST anomalies forcing minus CTRL with negative Atlantic Niño SST anomalies forcing (CTRL+ minus CTRL-), ensemble-mean of NTOP+ minus NTOP- respectively. **d-f** Same with **a-c** except for the differences between the topography removal experiment with positive Atlantic Niño SST anomalies forcing (NTOP+) and the topography removal experiment without Atlantic Niño SST anomalies forcing (NTOPclim) multiplied by the factor of 2, after excluding the differences between CTRL+ and CTRL- multiplied by the factor of 0.5 (2*[(NTOP+ minus NTOPclim) – (CTRL+ minus CTRL-)/2]) described in the Supplementary Discussion. The colors and vectors indicate statistical significance at the 10% level based on a two-sided Student's t-test. Source data are provided as a Source Data file in the Zenodo database.

Supplementary Tables

Supplementary Table 1 Observation and reanalysis for lag-regression.

Datasets	Variables	Periods	Temporal resolution	Horizontal resolution	Vertical levels
**HadISST**	sea surface temperature	1959-2021	monthly	1° x 1°	none
**ERA5 Reanalysis**	horizontal and vertical wind vectors	1959-2021		0.25° x 0.25°	1000, 975, 950, 925, 900, 875, 850, 800, 750, 700, 650, 600, 550, 500, 450, 400, 350, 300, 250, 200, and 175 hPa
**ERA-Interim Reanalysis**		1979-2018		0.70° x 0.70°	
**JRA-55 Reanalysis**		1959-2021		1.25° x 1.25°	

Supplementary Table 2 Years (January-December) used for lag-regression.

No pre-existing El Niño-Southern Oscillation years for lag-regression (44 years)
1960, 1961, 1962, 1963, 1964, 1965, 1967, 1968, 1969, 1970, 1972, 1975, 1977, 1978, 1979, 1980, 1981, 1982, 1984, 1986, 1988, 1990, 1991, 1993, 1994, 1995, 1996, 1997, 2001, 2002, 2003, 2004, 2005, 2006, 2007, 2009, 2012, 2013, 2014, 2015, 2017, 2018, 2019, 2020

Supplementary Table 3 Simulations with and without Atlantic Niño Sea Surface Temperature anomalies forcing

Experiment Name	Tropical Atlantic	Maritime Continent Land grid-point	Number of members	Integration Length	Initial Condition
**CTRL+/-**	Model climatology Sea Surface Temperature (SST) plus +/- Atlantic Niño SST anomalies	Realistic topography	60/60	1yr (from Jan)	Branch from the year 2006 of the PI-CTRL simulation
**NTOP+/-**		Flatten topography	60/60		
**NFRC+/-**		Flatten topography and reduced land friction	60/60		
**NTOPclim**	No Atlantic Niño sea surface temperature	Flatten topography	60	1 yr (from Jan)	

Supplementary Discussion

To validate the weak nonlinearity assumption, we conduct a new ensemble of 60 runs, which is identical to the NTOP ensemble except that the prescribed Atlantic Niño sea surface temperature (SST) forcing was removed. We refer to this new ensemble as NTOPclim. We evaluate the degree of nonlinearity in [(NTOP+)-(NTOP-)] - [(CTRL+)-(CTRL-)] by comparing its results shown in Fig. 2d-f (also in Supplementary Fig. 14a-c for easy comparisons) to those obtained by 2*[((NTOP+) - (NTOPclim)) - ((CTRL+) - (CTRL-))/2] as shown in Supplementary Fig. 14d-f. If the system were strictly linear, the results from these two analyses would be identical and both would show an eastward shifted La Niña-like response in the Pacific, because the climatological response to the topography removal would be completely canceled out. As can be seen in Supplementary Fig. 14, the response patterns between the two analyses are qualitatively similar, both show a reduced cooling (warm anomaly) in the western Pacific and an enhanced cooling (cold anomaly) in the eastern equatorial Pacific, particularly during August-September-October (ASO). Quantitively, however, there are some differences between these results in the sense that 2*[((NTOP+) - (NTOPclim)) - ((CTRL+) - (CTRL-))/2] produces a stronger and more consistent eastward shift of La Niña-like response after the MC topography is flattened. This result suggests that [(NTOP+)-(NTOP-)] - [(CTRL+)-(CTRL-)] may underestimate the eastward shift of La Niña-like response because of the relatively weak nonlinear interaction between the climatological state changes due to the MC topography removal and Atlantic Niño SST forced response. We further note that the results from 2*[((NTOP+) - (NTOPclim)) - ((CTRL+) - (CTRL-))/2] (Supplementary Fig. 14d-f) are more consistent with those in NFRC-NTOP shown in Fig. 2g-i. Therefore, we conclude that the relatively weak nonlinearity in [(NTOP+)-(NTOP-)] - [(CTRL+)-(CTRL-)] should not fundamentally alter the conclusion of the study. We expect a similar conclusion holds for NFRC-NTOP, as the response pattern is more robust.
